# Identification of the lipid biomarkers from plasma in idiopathic pulmonary fibrosis by Lipidomics

**DOI:** 10.1186/s12890-017-0513-4

**Published:** 2017-12-06

**Authors:** Feng Yan, Zhensong Wen, Rui Wang, Wenling Luo, Yufeng Du, Wenjun Wang, Xianyang Chen

**Affiliations:** 1 Department of Respiration, First Hospital of Tsinghua University, Beijing, 100016 China; 2 Division of Research and Education, First Hospital of Tsinghua University, Beijing, 100016 China; 3 Department of Neurology, The LongFu hospital of Beijing, Beijing, 100010 China; 4 Department of Gerontology, The First Hospital of ShanXi Medical University, Taiyuan, ShanXi, 030001 China; 5 Beijing Qiji Biotechnology Company, Beijing, 100193 China

**Keywords:** Idiopathic pulmonary fibrosis, Plasma, Lipid, Lipidomics, Biomarkers

## Abstract

**Background:**

Idiopathic pulmonary fibrosis (IPF) is an irreversible interstitial pulmonary disease featured by high mortality, chronic and progressive course, and poor prognosis with unclear etiology. Currently, more studies have been focusing on identifying biomarkers to predict the progression of IPF, such as genes, proteins, and lipids. Lipids comprise diverse classes of molecules and play a critical role in cellular energy storage, structure, and signaling. The role of lipids in respiratory diseases, including cystic fibrosis, asthma and chronic obstructive pulmonary disease (COPD) has been investigated intensely in the recent years. The human serum lipid profiles in IPF patients however, have not been thoroughly understood and it will be very helpful if there are available molecular biomarkers, which can be used to monitor the disease progression or provide prognostic information for IPF disease.

**Methods:**

In this study, we performed the ultraperformance liquid chromatography coupled with quadrupole time of flight mass spectrometry (UPLC-QTOF/MS) to detect the lipid variation and identify biomarker in plasma of IPF patients. The plasma were from 22 IPF patients before received treatment and 18 controls.

**Results:**

A total of 507 individual blood lipid species were determined with lipidomics from the 40 plasma samples including 20 types of fatty acid, 159 types of glycerolipids, 221 types of glycerophospholipids, 47 types of sphingolipids, 46 types of sterol lipids, 7 types of prenol lipids, 3 types of saccharolipids, and 4 types of polyketides. By comparing the variations in the lipid metabolite levels in IPF patients, a total of 62 unique lipids were identified by statistical analysis including 24 kinds of glycerophoslipids, 30 kinds of glycerolipids, 3 kinds of sterol lipids, 4 kinds of sphingolipids and 1 kind of fatty acids. Finally, 6 out of 62 discriminating lipids were selected as the potential biomarkers, which are able to differentiate between IPF disease and controls with ROC analysis.

**Conclusions:**

Our results provided vital information regarding lipid metabolism in IPF patients and more importantly, a few potentially promising biomarkers were firstly identified which may have a predictive role in monitoring and diagnosing IPF disease.

**Electronic supplementary material:**

The online version of this article (10.1186/s12890-017-0513-4) contains supplementary material, which is available to authorized users.

## Background

Idiopathic pulmonary fibrosis (IPF) is a disease featured as chronic, progressive, irreversible interstitial pneumonia with a poor prognosis of unknown etiology [1] and a median survival of 3 to 5 years after initial diagnosis [2, 3]. It more commonly occurs in patients of 50 to 70 years of age. This disease is characterized by the histological pattern of usual interstitial pneumonia [4, 5] with parenchymal fibrosis and excess collagen deposition [6]. It is well accepted that the pathology of this disease includes fibroblast/myofibroblast proliferation, activation of alveolar epithelial cells, with exacerbated deposit of extracellular matrix leading to the gradual destruction of the lung tissue [7]. Although there have been increasing number of studies investigating the pathogenesis of idiopathic pulmonary fibrosis (IPF), the lacking of effective treatments and early diagnostic tools indicate the urgent needs for reliable biomarkers in both diagnosing at early stage and monitoring the progression of this disease. In the recent publications, a few potentially useful blood cellular and molecular biomarkers have been identified, including chemokines, proteases and growth factors [8]. Despite the recent progress, there has not been a single biomarker proved to be useful in diagnosing and monitoring the progress of IPF. The diagnosis of IPF has not changed much over the last few years, which means that a multi-disciplinary approach is probably required for a breakthrough. With the new emergent technology of lipidomics, to identify novel lipid biomarkers for diagnostic and monitor purposes of IPF becomes possible.

Lipids comprise diverse classes of molecules that play a critical role in cellular energy storage, structure, and signaling [9–11]. Previously, lipids are only considered as the components of membranes and source. Now lipids are known to act as a indispensable factor in the immune response by organizing signaling complexes in cellular membrane, such as lipid rafts [12] or affecting the immune reaction by release of lipid-derived mediators [13, 14]. The etiologies of certain diseases have been proven to be associated with individual lipid molecules and many studies have indicated that certain lipid metabolic disorders or abnormalities can lead to a variety of human diseases [10, 15–19].

The role of lipids in lung and respiratory diseases has attracted more attention in recent years including cystic fibrosis, asthma and COPD which are all associated with abnormal metabolism. For example, the epithelium lipid metabolism has been proved to be changed in asthmatic patients [20, 21]. The amount of ceramides in the airway epithelium of a guinea pig model was found to be increased in response to the induction of experimental allergic asthma [22]. In another study, the increased amount of ceramides was detected in the airway epithelium and has been linked to cell death, infection susceptibility and immune inflammation in cystic fibrosis [23]. When it comes to IPF however, the human plasma lipid profiles of IPF is so far poorly understood and to identify reliable and unique lipid molecular biomarkers will be very beneficial in IPF diagnosis and management [24]. Based on the above reasons, to analyze and identify potential IPF-specific lipid biomarkers will contribute significantly to the diagnosis and management in IPF patients.

With the development of omicis [25] and the advanced mass spectrometry have made it feasible to identify and quantify a variety of lipids species in human samples such as the tandem mass spectrometer utilized (MS/MS) [26, 27],direct infusion MS (DIMS) [28], and liquid chromatography-mass spectrometry (LC-MS) [29–32]. Among different LC-MS platforms, ultraperformance liquid chromatography coupled with quadrupole time of flight mass spectrometry (UPLC-QTOF/MS) is widely adapted to lipidomics due to its enhanced reproducibility of retention time [33–35]. In this study, a global lipid profiling was performed containing measurement of 20 kinds of fatty acid, 159 kinds of glycerolipids, 221 kinds of glycerophospholipids, 47 kinds of sphingolipids, 46 kinds of sterol lipids, 7 kinds of prenol lipids, 3 kinds of saccharolipids, and 4 kinds of polyketides. Subsequently, the correlation analysis, receiver operating characteristic (ROC) analysis and orthogonal partial least squares discriminant analysis (OPLS-DA) were performed to evaluate the variations in lipid metabolites. The potential influence of gender, smoking history, and disease stages on the lipid metabolites was also looked at between IPF object and/or controls. Our results provided vital information regarding lipid metabolism in IPF patients and more importantly, a few potentially promising biomarkers were firstly identified which may have a predictive role in monitoring and diagnosing IPF disease.

## Methods

### Sample and collection

In this study, 22 IPF patients and 18 controls were obtained from the First Hospital of Tsinghua University from January 2014 to March 2016. All IPF patients were diagnosed with IPF after the age of 60, and all of controls were all above 60 years old when examined. The demographic figures are listed in the Table 1. The stages of disease are classified into mild and severe according to the arterial blood oxygen partial pressure (≧ 60 mmHg). The diagnosis of IPF patients was made according to the internal recommendations of the ATS/ERS/JRS/ALAT statement using high-resolution computed tomography (HRCT), as well as the clinical history of the patient [36]. All cases were discussed in our discussion team about interstitial lung disease composed of: a specialist in pulmonary rehabilitation, a specialist in occupational medicine, a radiologist, a rheumatologist, a pulmonologist and a pathologist. Blood samples were collected from each patient before received treatment and control. The controls were precluded if there was a history of pulmonary disease. For each subject, 10 ml whole blood was collected into a vessel tube containing heparin as anticoagulant. Each sample was centrifuged at 1500 x g for 15 min to collect serum and stored at -80 °C immediately until further analysis.

**Table 1:** Sample demographics of the study subjects

	IPF (*N* = 22)	Control (*N* = 18)
Age, mean	73	72
Gender (males/females)	11/11	8/10
Smoking History (Yes/No)	9/13	10/8
Stages (mild/severe)	11/11	–

### Lipidomics

Liquid chromatography-mass spectrometry (LC-MS)-grade isopropanol, acetonitrile, methanol and water were purchased from Fisher Scientific (New Jersey, USA). Debrisoquine, Pro-Asn, glycoursodeoxycholic acid andmalic acid, 4-nitrobenzoic acid (4-NBA) were products of Sigma (St. Louis, MO, USA). High purity formic acid (99%) was provided by Thermo-Scientific (Rockford, IL). The serum lipid extraction was conducted as described previously [37]. Briefly, the plasma samples were thawed on ice before being vortexed. For metabolite extraction, 25 μL of plasma sample was mixed with 175 μL of extraction buffer (25% acetonitrile in 40% methanol and 35% water). The sample was then incubated on ice for 10 min before centrifuged again at 14,000 rpm at 4 °C for 20 min. Subsequently, the produced supernatant was transferred to a fresh tube and dried under vacuum. After drying, the dried samples were reconstituted in 200 μL of buffer containing 5% methanol, 1% acetonitrile and 94% water. Fine particles were removed by centrifuge at 13,000 rpm for 20 min at 4 °C. Finally, the supernatant was transferred to a glass vial for ultraperformance liquid chromatography-quadrupole time-of-flight mass spectrometry (UPLC-QTOF-MS) analysis.

### Statistical analysis

All statistical analyses were conducted using R software version 2.9.1. The OPLS score plots and T-test and variable importance for projection (VIP) statistics were used to select significant variables leading to group separation. A supervised OPLS analysis was applied in our study to identify potential lipids that were used to classify the samples and remove non-correlated variables. The differences between the intensities of lipids in IPF and healthy controls were compared by T test when the data follow a normal distribution or Wilcoxon rank-sum test when otherwise. In this study, the identified lipids were pre-selected as potential biomarkers when VIP value is bigger than 1.0. To analyze the diagnostic value of potential lipid biomarkers for identifying IPF disease, a ROC analysis was performed. Correlation analysis of differential lipids was performed by MetaboAnalyst software. The influence of gender, smoking history, and stages of disease of individual subjects on lipid metabolism were evaluated by the Mann-Whitney *U*-test. The *p* values of less than 0.05 was considered statistically significant.

### Ethics statement

The clinical IPF samples included in this study were collected from the First hospital of Tsinghua University. All patient data were anonymous, so informed consent for participation was not required. The use of these samples was approved by the Institutional Review Board for human studies at the First Hospital of Tsinghua University. In this study, all personal information including name, date of birth, and contact information was all de-identified and not disclosed.

## Results

### IPF plasma lipid profiles of 507 apparent lipid species

In this study, the plasma lipid profiles of 507 individual lipid species were determined with lipidomics from 40 plasma samples; 22 from IPF patients with 18 controls (Additional file 1: Table S1). The detected individual plasma apparent lipid species were classified into 8 categories: fatty acid, glycerolipid, glycerophospholipid, sphingolipid, sterol lipid, prenol lipid, saccharolipid, and polyketide in accordance with NIH-funded Consortium, which has built an ongoing website tools offering precise information based on numerous lipidomics studies [38]. The 507 individual lipid species included 20 kinds of fatty acyls (3.94%), 159 kinds of glycerolipids (31.36%), 221 kinds of glycerophospholipids (43.59%), 47 kinds of sphingolipids (9.27%), 46 kinds of sterol lipids (9.07%), 7 kinds of prenol lipids (1.38%), 3 kinds of saccharolipids (0.59%), and 4 kinds of polyketides (0.79%), respectively. The significant differences of each individual apparent lipid species between IPF patients and control groups were detailed in the context of this study.

### Statistical analysis of the lipid profiling

**Fig. 1:**
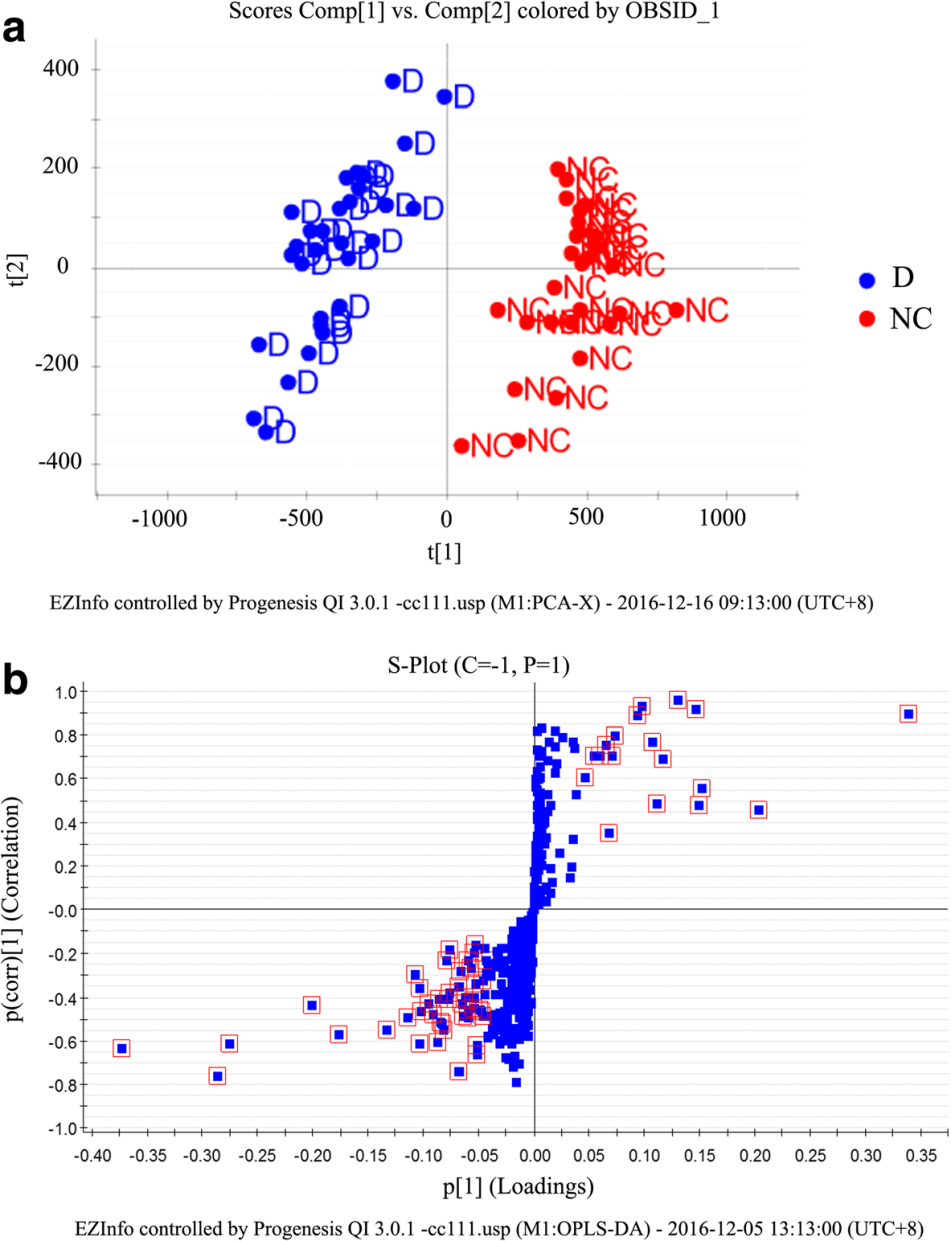
**a**. OPLS-DA scores plot based on the plasma lipid profiling of IPF patients (●D) and controls (●NC). **b**. S-plot used in the lipid biomarkers selection. The lipids marked (□) are the lipids selected as potential biomarkers

Supervised orthogonal partial least squares (OPLS) analysis identifed the biggest variation in lipid profiling using a few orthogonal latent variables and was performed with the lipid-obtained data on the plasma in positive ion mode by the UPLC-QTOF-MS/MS. The metabolic patterns were plotted by the OPLS-DA model (Fig. 1A). The OPLS-DA model was used to unfold the difference of plasma metabolic pattern between IPF patients and control group. The OPSL score plot revealed the significant deviation between IPF patients and controls.

In order to identify the potential biomarkers, the S-plot analysis was used based on the plasma lipid profiling data (Fig. 1B). By comparing changes in the lipid metabolite levels of IPF patients, a total of 62 unique lipids were detected [variable importance for projection (VIP > 1), Table 2]. The lipids were identified according to the reported methods as described previously [37]. They included 24 kinds of glycerophoslipids, 30 kinds of glycerolipids, 3 kinds of sterol lipids, 4 kinds of sphingolipids and 1 kind of fatty acid. The above lipids were selected as potential biomarkers from the S-plot for further analysis in our study.

**Table 2:** List of assigned statistically significantly lipid molecules and change trend of biomarkers on plasma lipid profile in positive ion mode after comparison of IPF patient samples to control samples

No.	Code	Calc m/z	t_R_	VIP	Trend^a^	Accepted Description	LipidIon	Element	Lipid class	*P*-Value
1	R1	1516.124	5.06	8.58719	↓	CL(1′-[20:0/20:0],3′-[18:1/18:2])	M + H,M + Na,M + H + Na	C_85_H_160_O_17_P_2_	Glycerophospholipids	7.90E-08^***^
2	R2	872.769	14.54	7.63644	↑	TG(16:0/18:2/18:2)[iso3]	M + H,M + NH_4_	C_55_H_98_O_6_	Glycerolipids	1.24E-07^***^
3	R3	806.567	4.52	6.33481	↓	PC(22:6/16:0)	M + H,M + Na,M + K,2 M + H,2 M + Na	C_46_H_80_NO_8_P	Glycerophospholipids	9.24E-07^***^
4	–	877.724	14.55	6.05829	↓	TG(16:1/18:1/18:2)[iso6]	M + Na,M + K,2 M + Na	C_55_H_98_O_6_	Glycerolipids	0.2370
5	–	874.785	14.82	4.60354	↑	TG(16:0/18:1/18:2)[iso6]	M + H-H_2_O,M + H,M + NH_4_,M + Na, M + K,2 M + NH_4_,2 M + Na	C_55_H_100_O_6_	Glycerolipids	0.1117
6	R4	782.567	4.23	4.39906	↓	PA(17:2/22:1)	M + ACN + H	C_42_H_77_O_8_P	Glycerophospholipids	0.0074^**^
7	–	903.739	14.55	3.8738	↓	TG(16:1/18:1/20:3)[iso6]	M + Na,M + K	C_57_H_100_O_6_	Glycerolipids	0.0017^**^
8	R5	900.801	14.84	3.45942	↑	TG(16:0/18:1/20:3)[iso6]	M + NH_4_,M + Na	C_57_H_102_O_6_	Glycerolipids	0.0174^*^
9	R6	902.816	15.11	3.44338	↑	TG(16:0/18:3/20:0)[iso6]	M + NH_4_,M + Na,2 M + Na	C_57_H_104_O_6_	Glycerolipids	0.0035^**^
10	R7	666.618	14.64	3.39521	↑	Stigmasteryl ester(16:1)	M + H,M + NH_4_,M + K,2 M + NH_4_,2 M + Na	C_45_H_76_O_2_	Stero lipids	7.79E-08^***^
11	R8	834.598	5.99	3.14588	↓	PS(20:0/19:0)	M + H-2H_2_O,M + H,M + Na,2 M + H,2 M + Na	C_45_H_88_NO_10_P	Glycerophospholipids	0.0019^**^
12	R9	369.352	14.65	3.00377	↑	(E,E)-3,7,11-Trimethyl-2, 6,10-dodecatrienyl dodecanoate	M + H-2H_2_O	C_27_H_48_O_2_	Fatty acids	7.31E-08^***^
13	R10	577.519	15.08	2.65073	↑	DG(16:0/18:1/0:0)	M + H-H_2_O	C_37_H_70_O_5_	Glycerolipids	7.02E-08^***^
14	R11	848.769	14.77	2.49731	↑	TG(16:0/16:1/18:1)[iso6]	M + H,M + NH_4_,M + Na,M + K	C_53_H_98_O_6_	Glycerolipids	0.0174^*^
15	R12	780.552	4.07	2.47828	↓	PE(20:0/17:2)	M + Na	C_42_H_80_NO_8_P	Glycerophospholipids	0.0232^*^
16	R13	603.535	15.07	2.44092	↑	DG(O-16:0/18:1)	M + Na	C_37_H_72_O_4_	Glycerolipids	7.02E-08^***^
17	–	758.569	3.96	2.34541	↓	PC(16:0/18:2)	M + H	C_42_H_80_NO_8_P	Glycerophospholipids	0.5407
18	R14	701.558	3.58	2.32011	↓	SM(d16:1/18:1)	M + H,M + Na,M + K,2 M + H,2 M + Na	C_39_H_77_N_2_O_6_P	Sphingolipids	0.0006^***^
19	R15	496.339	0.75	2.29702	↓	PC(0:0/16:0)	M + H-H_2_O,M + H,M + Na,M + K,2 M + K	C_24_H_50_NO_7_P	Glycerophospholipids	0.0049^**^
20	R16	369.352	15.00	2.27907	↑	3-Deoxyvitamin D3	M + H,M + NH_4_,2 M + H	C_27_H_44_	Stero lipids	7.31E-08^***^
21	–	870.753	14.27	2.16619	↓	TG(14:1/16:0/22:4)[iso6]	M + NH_4_,M + Na,M + K	C_55_H_96_O_6_	Glycerolipids	0.2829
22	R17	925.796	14.55	2.1151	↑	TG(18:4/20:3/22:0)[iso6]	M + H-2H_2_O	C_63_H_108_O_6_	Glycerolipids	7.86E-08^***^
23	R18	801.682	10.78	2.06029	↓	SM(d17:1/24:0)	M + H,M + Na	C_46_H_93_N_2_O_6_P	Sphingolipids	1.85E-05^***^
24	–	899.709	13.99	1.99376	↓	TG(16:1/18:2/20:4)[iso6]	M + NH4,M + Na,M + K	C_57_H_96_O_6_	Glycerolipids	0.1458
25	R19	546.352	1.24	1.88084	↓	PS(22:0/0:0)	M + H-2H_2_O	C_28_H_56_NO_9_P	Glycerophospholipids	0.0249^*^
26	–	873.693	13.97	1.87046	↓	TG(15:1/17:1/20:4)[iso6]	M + Na,M + K	C_55_H_94_O_6_	Glycerolipids	0.1245
27	–	834.598	5.14	1.83438	↓	PC(22:4/18:2)	M + H,M + Na,2 M + Na	C_48_H_84_NO_8_P	Glycerophospholipids	0.4226
28	R20	832.580	5.49	1.81376	↓	PC(20:2/18:2)	M + Na,2 M + Na	C_46_H_84_NO_8_P	Glycerophospholipids	1.22E-06^***^
29	–	931.771	15.01	1.81008	↓	TG(17:0/17:0/22:5)[iso3]	M + Na	C_59_H_104_O_6_	Glycerolipids	0.5582
30	–	786.601	4.84	1.76508	↓	PC(18:1/18:1)	M + H-H_2_O,M + H,M + K	C_44_H_84_NO_8_P	Glycerophospholipids	0.9026
31	–	1564.121	2.72	1.68796	↓	PC(22:4/14:0)	M + NH_4_,M + K,2 M + H	C_44_H_80_NO_8_P	Glycerophospholipids	0.2314
32	R21	603.532	14.82	1.64593	↑	DG(18:0/18:2/0:0)[iso2]	M + H-H_2_O	C_39_H_72_O_5_	Glycerolipids	7.53E-08^***^
33	R22	575.503	14.54	1.62251	↑	DG(16:0/18:2/0:0)[iso2]	M + H-H_2_O	C_37_H_68_O_5_	Glycerolipids	2.58E-07^***^
34	–	812.614	6.86	1.61868	↓	PS(O-20:0/20:2)	M + H-H_2_O,M + H	C_46_H_88_NO_9_P	Glycerophospholipids	0.0893
35	–	849.693	14.21	1.61511	↓	TG(14:1/18:1/18:2)[iso6]	M + NH_4_,M + Na,M + K	C_53_H_94_O_6_	Glycerolipids	0.2829
36	–	904.831	15.36	1.53098	↑	TG(17:0/18:1/19:1)[iso6]	M + NH_4_,M + Na,M + K,2 M + Na	C_57_H_106_O_6_	Glycerolipids	0.1615
37	R23	828.551	5.01	1.5305	↓	PS(P-18:0/19:0)	M + H-H_2_O,M + K	C_43_H_84_NO_9_P	Glycerophospholipids	0.0428^*^
38	R24	599.504	14.54	1.49112	↑	DG(18:1/18:3/0:0)[iso2]	M + H-H_2_O,M + H	C_39_H_68_O_5_	Glycerolipids	1.21E-07^***^
39	–	901.725	13.34	1.48742	↓	TG(16:0/16:0/22:6)[iso3]	M + NH_4_,M + Na	C_57_H_98_O_6_	Glycerolipids	0.7340
40	R25	955.759	13.01	1.48372	↓	TG(19:1/22:6/22:6)[iso3]	M + H-2H_2_O	C_66_H_102_O_6_	Glycerolipids	3.56E-06^***^
41	–	925.724	14.16	1.46671	↓	TG(12:0/22:2/22:6)[iso6]	M + NH_4_,M + Na,M + K	C_59_H_98_O_6_	Glycerolipids	0.1458
42	–	879.739	13.99	1.38113	↓	GalCer(d18:1/26:1(17Z))	M + ACN + H	C_50_H_95_NO_8_	Sphingolipids	0.7340
43	R26	601.518	14.53	1.36641	↑	DG(18:1/18:2/0:0)[iso2]	M + H-H_2_O	C_39_H_70_O_5_	Glycerolipids	8.04E-07^***^
44	–	810.599	4.57	1.32667	↓	PC(18:0/18:1)	M + Na	C_44_H_86_NO_8_P	Glycerophospholipids	0.1534
45	R27	759.637	6.96	1.30466	↓	SM(d18:1/20:0)	M + H,M + Na,2 M + H	C_43_H_87_N_2_O_6_P	Sphingolipids	0.0139^*^
46	–	524.371	1.10	1.29548	↓	PC(18:0/0:0)	M + H,M + K	C_26_H_54_NO_7_P	Glycerophospholipids	0.2479
47	R28	830.566	3.80	1.25023	↓	PC(22:6/18:2)	M + H,M + Na	C_48_H_80_NO_8_P	Glycerophospholipids	0.0038^**^
48	R29	1620.182	4.50	1.22041	↓	PI-Cer(d20:0/16:0)	M + H-2H_2_O,M + K,2 M + H	C_42_H_84_NO_11_P	Glycerophospholipids	7.90E-08^***^
49	–	828.551	4.04	1.20343	↓	PE(22:6/19:0)	M + Na	C_46_H_80_NO_8_P	Glycerophospholipids	0.2060
50	R30	948.800	14.59	1.20004	↑	TG(18:1/20:3/20:4)[iso6]	M + NH_4_	C_61_H_102_O_6_	Glycerolipids	7.90E-08^***^
51	R31	794.602	4.86	1.19345	↓	PC(20:3/P-18:1)	M + H,M + Na,2 M + H	C_46_H_84_NO_7_P	Glycerophospholipids	1.93E-07^***^
52	R32	790.569	5.79	1.18604	↓	PA(20:5/20:1)	M + H-H_2_O,M + ACN + H	C_43_H_73_O_8_P	Glycerophospholipids	0.0012^**^
53	–	881.754	14.25	1.18421	↓	TG(15:0/18:1/19:1)[iso6]	M + Na	C_55_H_102_O_6_	Glycerolipids	0.2829
54	–	1494.138	6.25	1.17806	↓	CL(1′-[20:0/18:0],3′-[18:0/18:0])	M + H	C_83_H_162_O_17_P_2_	Glycerophospholipids	0.5751
55	R33	851.708	13.63	1.14176	↓	20:1-Glc-Sitosterol	M + H-H_2_O	C_55_H_96_O_7_	Stero lipids	0.0161^*^
56	R34	897.696	13.73	1.10216	↓	TG(18:3/18:3/20:5)[iso3]	M + H	C_59_H_92_O_6_	Glycerolipids	0.0014^**^
57	–	542.321	0.60	1.09805	↓	PC(20:5/0:0)	M + H	C_28_H_48_NO_7_P	Glycerophospholipids	0.3318
58	–	1576.219	8.50	1.05263	↓	PC(17:0/19:1)	M + K,2 M + H	C_44_H_86_NO_8_P	Glycerophospholipids	0.2471
59	–	951.740	14.31	1.04393	↓	TG(18:4/20:0/20:5)[iso6]	M + Na,M + K	C_61_H_100_O_6_	Glycerolipids	0.3918
60	–	896.768	14.27	1.01614	↓	TG(18:1/18:2/18:3)[iso6]	M + NH_4_,M + Na	C_57_H_98_O_6_	Glycerolipids	0.9892
61	R35	920.768	14.30	1.01291	↑	TG(16:0/20:3/20:5)[iso6]	M + NH_4_,M + K	C_59_H_98_O_6_	Glycerolipids	1.40E-06^***^
62	–	905.754	13.68	1.00186	↓	TG(12:0/20:1/22:3)[iso6]	M + Na	C_57_H_102_O_6_	Glycerolipids	0.8811

^a^Change trend of lipids on IPF patients vs controls. The levels of potential lipid biomarkers labeled with (↑) and (↓) represent up-regulation and down-regulation, respectively. The variable importance for projection (VIP) statistics and T-test were used applied to select significant variables leading to group separation. VIP value larger than 1.0 were considered statistically significant and significant differences in statistics were also defined by P values of < 0.05 (*), < 0.01 (**), and < 0.001 (***)

All the determined glycerophospholipids and sphingolipids showed a decreasing tendency in IPF objects (Table 2). Although all of glycerophospholipids and sphingolipids also showed a similar decreasing pattern in IPF patients, the magnitude of their drop was not the same. 15 out of 30 glycerolipids and 1 out of sterol lipids were lower in IPF objects. On the other hand, the remaining glycerolipids, sterol lipids and fatty acids had an increased level in IPF objects. These findings suggested that the observed changes in lipid profiles were likely caused by different expression in IPF patients.

### Correlation and receiver operating characteristic (ROC) curve analysis

**Fig. 2:**
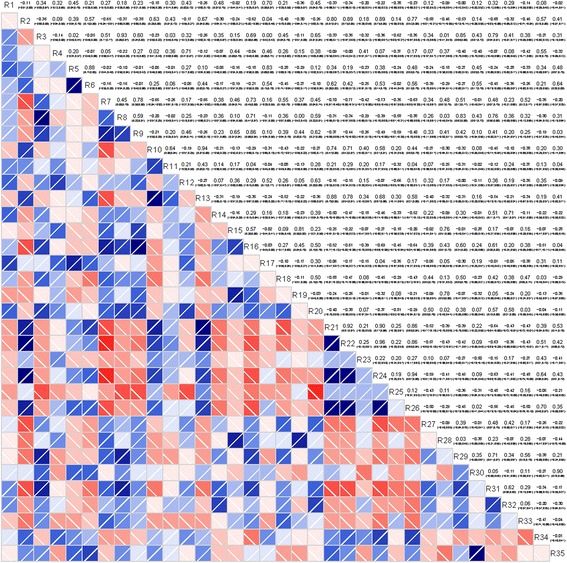
Correlation analysis of the 35 pre-selected discriminating lipids in IPF patients and controls. R1 to R35 represents the corresponding pre-selected discriminating lipids as shown in Table 2. Red and blue represent a negative and positive correlation, respectively. The color depth represents the degree of correlation: the deeper color indicates higher correlation

To better understand the relationship of metabolite differences with IPF disease, correlation analysis was applied to analyze these identified lipids data (Fig. 2). Firstly, The differences between the intensities of 62 identified lipids in IPF and healthy controls were compared by T test when the data follow a normal distribution or Wilcoxon rank-sum test when otherwise. Comprehensive analysis with *P*-value, 35 out of 62 identified lipids were selected to further analyze (Table 2, R1 to R35). Then, we evaluated the correlation between IPF disease and 35-selected lipids. As shown in Fig.2, the close correlations can be detected between IPF disease and 12 out of 35 identified lipids, including TG(16:0/18:2/18:2)[iso3] (R2), PC(22:6/16:0) (R3), Stigmasteryl ester(16:1) (R7) and (E,E)-3,7,11-Trimethyl-2,6,10-dodecatrienyl dodecanoate (R9), DG(O-16:0/18:1) (R13), 3-Deoxyvitamin D3 (R16), TG(18:4/20:3/22:0)[iso6] (R17), DG(18:0/18:2/0:0)[iso2] (R21), DG(16:0/18:2/0:0)[iso2] (R22), DG(18:1/18:3/0:0)[iso2] (R24), TG(19:1/22:6/22:6)[iso3] (R25), and DG(18:1/18:2/0:0)[iso2] (R26) (correlation absolute value > 0.7). This result indicates that these individual lipid molecules potentially are useful to differentiated the IPF patients from control group and the accuracy and efficiency possibly can be further increased if more lipid candidates are to be used.

**Fig. 3:**
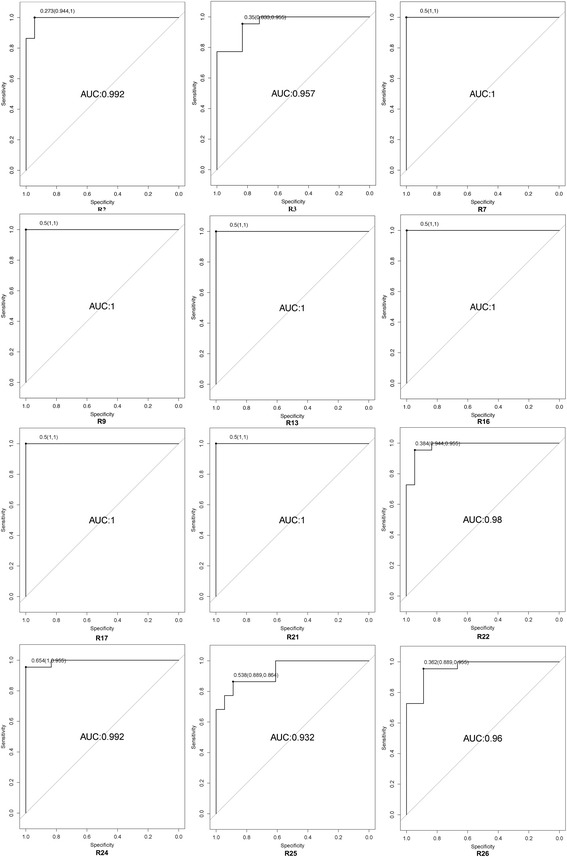
ROC curves analysis of 12 lipid metabolite for discriminating IPF objects from controls

Theoretically, those lipid molecules possessing close correlation with IPF disease would be more promising to be used as biomarkers. To further demonstrate the ability of the 12 out of 35 identified lipids to identify the IPF objects and controls, the receiver operating characteristic (ROC) curve was applied according to the results for the area under the curve (AUC) and sensitivity/specificity at the best cut-off points (Fig. 3). The AUC values of these molecules showed significant differences in the discovery set. 6 (R7, R9, R13, R16, R17 and R21) of them showed higher sensitivity/specificity for identifying IPF objects from controls (Fig. 3). This result have demonstrated that each of the sixmolecules may be used as potential biomarkers in diagnosing IPF disease in the future.

### Gender, smoking, and disease status-associated differences in the lipid levels of six-promising biomarkers

To demonstrate whether gender, smoking history, and disease stage have influence on selecting useful future biomarkers, 6 lipid levels in serums from IPF objects and control were further studied. Table 3 summarises the significance of all 6 biomarkers with regards to gender, smoking history, and disease stage-associated differences. None of these 6 was found to have significant differences related to these parameters mentioned above.

**Table 3:** Differences in the lipid levels of eight-promising biomarkers under the influence of gender, smoking, and disease status. The statistical significance was determined by Mann-Whitney *U*-test

No.	Gender/*P*-value	Smoking/P-value	Disease status/*P*-value
R7	0.92	0.92	0.65
R9	0.51	0.51	0.91
R13	0.81	0.16	0.17
R16	0.40	0.40	0.84
R17	0.85	0.43	0.30
R21	0.96	0.17	0.15

## Discussion

IPF is a kind of chronic and progressive disease with low survival rate, and remains to be a clinical challenge. It is regarded as a fetal disease due to its poor prognosis and a low median survival of only 3~5 years after diagnosis. At the moment, only clinical data are available for diagnosis to researchers and clinicians, which is of limited value as they do not reflect the precise pathological mechanisms underlying IPF. To better elucidate disease mechanism and make early diagnosis of IPF, identification of molecular biomarkers with high diagnostic value is paramount. In recent publications, a number of biomarkers have been hypothesized to be present in serum used as potential prognostic or diagnostic tools. The main protein biomarkers associated with cell dysfunction are the Krebs con den lungen-6 (KL-6) antigen and the surfactant protein A and protein D (SP-A and SP-D) [39]. Biomarkers found in IPF involved in fibrogenesis are matrix metalloproteinases-1 and -7 (MMP-1 and MMP-7), which play a role in the breakdown and remodeling of extracellular matrix components [40]. However, no lipid biomarkers have yet been studied for IPF. This work is the first comprehensive investigation into the potential lipid biomarkers for IPF using UPSL-QTOF-MS/MS.

Lipids is the fundamental component of cellular membranes, also exert several essential and critical roles in cellular functions including energy storage, signal transduction, formation of membrane bilayer and cellular barriers. The metabolism of lipid is indicated in numerous human diseases, such as Alzheimer’s disease, diabetes, obesity, atherosclerosis and several types of respiratory diseases. There are eight major categories of lipid types based on their structures: fatty acid, glycerolipid, saccharolipid, polyketide, sphingolipid, sterol lipids, prenol lipid and glycerophospholipid. Although abnormal lipid metabolism has been shown to result in cystic fibrosis and lung injury published by Ollero’s and Goss’ research groups, respectively [41, 42], its potential role in pathology of IPF remains unclear.

In this study, analysis of lipid profile from 22 IPF patients and 18 control subjects revealed the characterization of lipid composition. The glycerophospholipids (GPs) appears to be the important biological molecules for the backbone of cellular membranes. Besides an integral component of biomembranes, GPs also seem to be a reservoir of a large amount of many bioactive mediators [43], which are produced by the reaction of phospholipases on GPs. GPs can be further divided into different categories including glycerophosphoglycerols (PGs), glycerophosphatidic acids (PAs), glycerophosphoinositols (PIs), glycerophosphoserines (PSs), glycerophosphoethanolamines (PEs) and cardiolipin (CLs).

In our study, 2 CLs, 2 PAs, 13 PCs, 2 PEs, 1 PIs, and 4 PSs were identified as unique lipids of IPF patients based on VIP scores (Table 2). The levels of all screened GPs was decreased in IPF patients compared to the control subjects. This phenomenon may be due to the fact that PG is a precursor of CL biosynthesis and PA is the critic substrate for biosynthesis of PI, PG, PE, and PC. A previous TLC-MALDI/TOF-based metabonomics study displayed significantly decreased plasma PCs in cystic fibrosis patients, including PC(P-40:1), PC(38:6), PC(38:5), PC(38:4), PC(38:2), PC(38:0) and PC(36:5) [44]. However, using electrospray ionization tandem mass spectrometry (ESI-MS/MS)-based metabonomics, Marien et al. showed an increase in non-small cell lung cancer of PI(38:3), PI(40:3), PI(38:2), PS(32:0), PS(36:4), PS(36:1), PS(40:2), PS(38:1), PS(34:0), PS(40:1), PS(38:5), PS(34:2) and PS(38:4), compared to normal lung tissues [45]. Increasing evidence implicates that GPs biomarkers were several in agreement with abnormal GPs metabolism found in above mentioned lung disease and IPF model. This result indicated that GPs with top scores could be used as potential biomarkers for IPF.

Our study identified 159 glycerolipids in IPF patients and 30 out of these 159 have the capacity to differentiate IPF patients from control subjects (VIP > 1, Table 2). Glycerolipids possess long chain fatty acids in ester linkage to the glycerol backbone and were classified into two major groups: diacylglycerol (DG) and triacylglycerol (TG), which are the most abundant lipids found in circulating plasma [46]. For example, all six DGs out of 30 glycerolipids showed similar tendencies to increase in the plasma from IPF patients with variable magnitudes. Interestingly, 9 out of 24 TGs displayed increased levels in IPF patients compared to control subjects. A previous study demonstrated that glycerolipids were significantly reduced in lung tissues of mice, which lacked the p53 oncogene [47]. This result indicates that the abnormal function of glycerolipid was associated with lung disease. Further investigation of the exact roles of glycerolipids in IPF patients would be beneficial.

We detected 47 sphingolipids (SLs) by UPSL-QTOF-MS/MS in plasma samples and found that 4 out of them can distinguish the IPF patients from control subjects. 4 types of SLs (GalCerd18:1/26:1, d18:1/20:0, d16:1/18:1, d17:1/24:0) significantly decreased in plasma of patients with IPF. SLs are ubiquitous cellular membrane components that are implicated in multiple cellular processes including autophagy, apoptosis, differentiation and cell division [48]. Ceramide is generated from either sohingomyelin or de novo sphingolipids synthesis [49] and its upregulation has been found in chronic-obstructive pulmonary disease [50]. In contrast, our results showed decreased SLs serum level in IPF patient, which could be due to the differences between different respiratory diseases. In the future, we will compare SLs levels in serum and bronchoalveolar lavage fluids between respiratory diseases, such as COPD and pneumonia. Although it remains to be equivocal whether differences exist in SLs levels between IPF and other respiratory diseases, our data have provided a direction for future investigation into implications of these SLs in IPF patients.

Moreover, this study identified 46 kinds of serol lipids and 20 kinds of fatty acids. 3 kinds of 46 serol lipids and 1 kind of 20 fatty acids were considered as potential biomarkers. Fatty acids are the most important class of lipids and function as precursors of various bioactive lipid molecules. In our study, fatty acid (E,E)-3,7,11-Trimethyl-2,6,10-dodecatrienyl dodecanoate was shown to be positively correlated with IPF and may distinguish IPF patients from control subjects. Three (3-Deoxyvitamin D3,16:1 Stigmasteryl ester, and 20:1-Glc-Sitosterol) out of 46 sterol lipids identified have been shown to correlate with the IPF. Other studies have shown that plasma cholesterol level was significantly increased in diet-induced hyperlipidomic rats [51], and cholesteryl ester apparent lipid molecular species have high sensitivity, specificity and accuracy in the diagnosis of prostate cancer [10]. Further validation of the specificity of these lipid molecules in IPF patients among respiratory disease would be desirable.

Furthermore, we investigated lipid molecules of 12 metabolites possessing close correlations with IPF disease. 6 of them showed higher sensitivity and specificity for identifying IPF patients from control subjects ROC analysis (Fig. 3). Previous study has demonstrated that age, gender, and smoking status can affect plasma lipid metabolite levels in healthy adults [52, 53]. The impact of gender and smoking on 6 promising biomarker levels were determined in this study and no correlation was found (Table 3). Further validations on whether the 6 identified promising biomarkers has the increased ability to discriminate IPF objects from healthy controls or other respiratory disease are highly suggested.

For the analysis of IPF patients and biomarkers, the potential defect in this study was the small sample size. Another limitation is the lack of a longitudinal study, which made it impossible to observe the clinical impact of the present discovery as no treatment responses can be assessed. Although we have identified promising lipid biomarkers in this study, further validations are necessary to evaluate the specificity of the identified biomarkers for IPF diagnosis. Then, further longitudinal multicenter studies would contribute more to evaluate the real value of lipid biomarkers as diagnostic and prognostic tools. In addition, the lipid biomarkers identified in this study should be compared with known diagnostic and prognostic biomarkers in the future, such as KL-6, SP-A, SP-D. This will provide an insight into a better understanding of the diagnostic and prognostic utility of identified lipid biomarkers. Lastly, more specific lipidomics analyses of these lipid changes in IPF will probably help to better understand the IPF pathology and may contribute to future development of novel therapeutic targets.

## Conclusions

In conclusions, our study has yielded important information regarding lipid metabolism in IPF patients and presents the first identification of promising potential biomarkers for the diagnosis of IPF. Our results demonstrate that individual lipid molecules have the ability to differentiate IPF from controls. Implications for future studies include validation of the accuracy of biomarkers to diagnose IPF and investigation in their IPF-specificity compared to other respiratory diseases, such as asthma, COPD, and infective pneumonia.

## Additional file

Additional file 1: Table S1. This file contains the plasma lipid profiles of 507 individual lipid species were determined with lipidomics from 40 plasma samples; 22 from IPF patients with 18 controls. (XLS 562 kb)

## Abbreviations

CLs: Cardiolipin
IPF: Idiopathic pulmonary fibrosis
OPLS-DA: Orthogonal partial least squares discriminant analysis
PAs: Glycerophosphatidic acids
PCs: Glycerophosphocholines
PEs: Glycerophosphoethanolamines
PGs: Glycerophosphoglycerols
PIs: Glycerophosphoinositols
PSs: Glycerophosphoserines
ROC: Receiver operating characteristic
UPLC-QTOF/MS: Ultraperformance liquid chromatography coupled with quadrupole time of flight mass spectrometry
VIP: Variable importance for projection
